# Differences in gut microbiota and fecal bile acids between Caucasian and Hispanic children and young adults with ulcerative colitis

**DOI:** 10.14814/phy2.15752

**Published:** 2023-06-21

**Authors:** Lara Aboud Syriani, Riddhi Parsana, Ramón A. Durazo‐Arvizu, Sonia Michail

**Affiliations:** ^1^ College of Osteopathic Medicine of the Pacific Western University of Health Sciences Pomona California USA; ^2^ Children's Hospital of Los Angeles Los Angeles California USA; ^3^ Keck School of Medicine University of Southern California Los Angeles California USA

## Abstract

Ulcerative Colitis (UC) is an inflammatory bowel disease (IBD) that has been associated with gut dysbiosis. Changes in the gut microbiome lead to changes in bile acids (BA) metabolism, which changes the BA profiles in patients with UC. We conducted this study to investigate the differences in bile acids and gut microbiota between Hispanic and Caucasian children and young adults with UC. Twenty‐seven Caucasian and 20 Hispanic children and young adults with UC were enrolled in the study. BAs were extracted from the subjects' stool samples and analyzed by liquid chromatography–mass spectrometry. Microbial DNA was also extracted from the stool samples to perform 16s rRNA amplicon sequencing. The median levels of cholic acid and taurolithocholic acid were found to be significantly higher in Hispanic children and young adults with UC compared to their Caucasian counterparts. The abundance of the gut microbiota that metabolizes BAs such as Proteobacteria, Pseudomonadaceae, *Pseudomonas*, *Ruminococcus gnavus*, and *Escherichia coli* were also all significantly higher in Hispanic children and young adults as well. The distinct BA profile that we found in Hispanic children and young adults with UC, in addition to the unique composition of their gut microbiome, provide them with a protective gut environment against inflammation, which is contrary to the common believe that Hispanics have worse IBD.

## 1 INTRODUCTION

Ulcerative colitis (UC) is an inflammatory bowel disease (IBD) characterized by chronic episodes of clinical remission interspersed with relapses of inflammatory activity in the gastrointestinal tract (Pascarella et al., 2009). IBD has been increasing dramatically in incidence and prevalence in pediatric populations worldwide (Kuenzig et al., 2022). IBD has been associated with gut dysbiosis (Duboc et al., 2013). Changes in intestinal microbiota, in turn, induce changes in bile acid (BA) metabolism leading to alterations in BA profiles in patients with UC (Duboc et al., 2013).

We designed this study to look more specifically at Hispanic and Caucasian children and young adults with mild to moderate UC who have similar demographics/characteristics, including age, Pediatric Ulcerative Colitis Activity Index (PUCAI) score, medications for UC treatment, and incidence of previous *Clostridioides difficile* infection. This design will allow us to investigate whether BAs and their metabolizing bacteria are different between the two groups while controlling for possible confounding variables.

In healthy individuals, cholic acid (CA) and chenodeoxycholic acid (CDCA) are primary bile acids (PBAs) synthesized from cholesterol in the liver and secreted into the duodenum following their conjugation with taurine or glycine groups (Cheng et al., 2019). Most of these BAs get reabsorbed in the ileum and returned to the liver through enterohepatic circulation (Gasaly et al., 2021). The rest of the BAs that make it to the colon either get metabolized by gut microbiota or get excreted with feces (Gasaly et al., 2021). Colonic bacteria normally perform deconjugation, dehydroxylation, oxidation and epimerization, esterification, and desulfatation reactions to convert PBA into secondary bile acids (SBA). Deoxycholic acid (DCA), lithocholic acid (LCA), and ursodeoxycholic acid (UDCA) are SBAs that are produced from those reactions.

It has been reported in the literature that patients with IBD usually have higher levels of PBAs and conjugated BAs in their fecal samples (Li et al., 2021). Previous studies have shown that changes in the bacterial metabolization of those BA could be contributing to those IBD‐associated BA changes (Li et al., 2021). In turn, the alterations in BA profiles can affect the gut microbiota composition, contribute to inflammation, and cause BA malabsorption associated with diarrhea (Li et al., 2021).

It is also well established that patients with IBD have reduced SBAs compared to healthy individuals (Duboc et al., 2013; Molinero et al., 2019). This shift is thought to be contributing to the inflammatory state in UC since SBAs play a role in anti‐inflammation (Duboc et al., 2013). Some of the intestinal bacterial taxa that play a role in transforming BAs have been found to have altered levels in IBD patients in multiple previous studies. *Ruminoccus gnavus* and *Escherichia coli* are among the most common species that are enriched in UC patients (Berry & Reinisch, 2013; Hall et al., 2017; Schirmer et al., 2019). The Ruminococcaceae family is known to have the ability to perform 7α‐dehydroxylation of CA and CDCA to generate SBAs (Sinha et al., 2020). While the *E*. *coli* species from the proteobacteria phylum can perform oxidation and epimerization reactions via hydroxysteroid dehydrogenase (HSDH) to convert PBAs to SBAs (Molinero et al., 2019).

IBD has been historically considered a disease that primarily affects Caucasians; however, recent trends have shown a continuous increase in IBD incidence among different racial and ethnic groups (Afzali & Cross, 2016). The rapidly shifting demographics in the United States and the expected increase in the Hispanic population by 2050 urge us to understand better how IBD may affect Hispanics living in the US, especially in the vulnerable pediatric populations (Afzali & Cross, 2016).

Literature investigating the differences in fecal BAs profiles and gut microbiota composition between Hispanic and Caucasian patients with UC is scarce. Despite the presence of some evidence in the literature suggesting that Hispanic UC patients are more likely to have worse disease extent and severity when compared to Caucasians (Afzali & Cross, 2016), our results demonstrate a novel finding of protective gut environment provided by significant increase in SBAs and the gut microbiota generating them in Hispanics.

## 2 MATERIALS AND METHODS

### 2.1 Patients and sample collection

This study was conducted under IRB# CCI‐11‐00148 at the Children's Hospital of Los Angeles. The investigator and study team recruited pediatric patients diagnosed with UC during their clinic visit, inpatient hospitalization, or through the department list between January 1, 2010, and March 1, 2020. After obtaining informed consent, subjects were asked to complete a questionnaire including gender, date of birth, race, weight, height, and medications. Subjects were also asked to provide one stool sample. There were 4/20 Hispanic subjects and 3/27 Caucasian subjects diagnosed with UC within a year of sample collection. Those stool samples were used to extract the following 15 BAs: CA, glycocholic acid (G‐CA), taurocholic (T‐CA), CDCA, glycochenodeoxycholic acid (G‐CDCA), taurochenodeoxycholic acid (T‐CDCA), DCA, glycodeoxycholic acid (G‐DCA), taurodexoycholic acid (T‐DCA), LCA, glycolithocholic (G‐LCA), taurolithocholic acid (T‐LCA), UDCA, glycoursodeoxycholic acid (G‐UDCA), tauroursodeoxycholic acid (T‐UDCA). All samples were immediately transported on ice and placed at −80°C for storage until the bile extraction procedure was performed at the University of California Davis/West Coast Metabolomics Center. Additionally, a food frequency questionnaire (Block et al., 1992; Block & Subar, 1992) was used to collect diet information from participating children and young adults at the time of stool collection. Forty‐seven patients were selected based on similarities in age, PUCAI scores, and *C*. *difficile* infection incidence history. Patients were divided into two groups based on self‐report of ethnicity: 27 Caucasian patients and 20 Hispanic patients. Both Hispanic and Caucasian subjects were born and living in the United States.

### 2.2 BA extraction procedure and analysis

Fifteen milligrams of aliquots from stool samples was placed in 2‐mL Eppendorf tubes on dry ice and kept frozen. A 10 μL of antioxidant solution (0.2 mg/mL solution butylated hydroxytoluene/ethylenediaminetetraacetic acid in 1:1 MeOH:water) was added. This was enriched with 10 μL of BA surrogate (SSTD, 1000 nM) and extracted with 500 μL of cold methanol and stainless‐steel grinding balls. The extract was homogenized using GenoGrinder 2 × 30 s, centrifuged, and the supernatant was transferred to a 1.5‐mL Eppendorf tube containing 10 μL 20% glycerol solution in MeOH. A second aliquot of 500 μL of cold methanol was added to the centrifugation pellet. This was homogenized again using GenoGrinder 2 × 30 s, centrifuged, and the second supernatant was combined with the first one in the 1.5‐mL Eppendorf tube. Then, the vials were transferred to Speed‐vac and evaporated to dryness. Dry samples were reconstituted for liquid chromatography–mass spectrometry in 100 μL of 1‐phenyl 3‐hexadecanoic acid urea/1‐cyclohexyluriedo‐3‐dodecanoic acid 100 nM/L in methanol/acetonitrile 50:50, vortexed for 10 s, then sonicated for 5 min. The rack of samples was set on wet ice for 15 min. Then, samples were centrifuged for 3 min at the highest speed, followed by transferring the supernatant to a glass insert in an amber HPLC vial. These were stored at −20°C until liquid chromatography–mass spectrometry analysis, which was carried out on a Thermo Vanquish UPLC/AB Sciex Qtrap with the targeted MRM method.

### 2.3 Microbial DNA extraction

DNA was extracted from stool samples using the QIAamp Power Fecal DNA kit (Qiagen). To mechanically lyse the fecal cells, a Vortex‐Genie 2 was used with a horizontal tube holder adaptor. NanoDrop was used to confirm the extracted DNA quantity and quality.

### 2.4 16s rRNA amplicon sequencing and data analysis

The procedure of 16s rRNA sequencing was previously described by Rotondo‐Trivette et al. (2021). Briefly, amplification of the 16s bacterial DNA V4 region from stool samples was done by PCR using the following barcoded primers: 515FB: 5′‐GTG YCA GCM GCC GCG GTA A‐3′; 806RB: 5′‐GGA CTA CNV GGG TWT CTA AT‐3′. Those primers were modified from the original 515F‐806R primer pairs. Each one of the PCR samples was run in a triplicate. Agilent High Sensitivity DNA Bioanalyzer chips were used to assess library qualities. The following custom sequencing primers were used to sequence all the samples: Read 1 (5′‐TAT GGT AAT TGT GTG YCA GCM GCC GCG GTA A‐3′), Read 2 (5′‐AGT CAG CCA GCC GGA CTA CNV GGG TWT CTA AT‐3′) and Index (5′‐AAT GAT ACG GCG ACC ACC GAG ATC TAC ACG CT‐3′). Lastly, Illumina MiSeq Reagen Kit v2 flowcell was used to perform paired‐end sequencing (2 × 150 bp) on an Illumina MiSeq System. The resulting amplicons were de‐multiplexed using QIIME version 1.9.1 (Caporaso et al., 2010) and processed an amplicon sequence variant table using DADA2 v1.5.2. Briefly, DADA2 performs quality filtering, de‐noising, sample inference, and chimera removal using an empiric error model to generate an exact amplicon sequence. The amplicon sequence variants were analyzed using CLC Genomic workbench version 20.0.4 (CLC; Bio‐Qiagen). Operational taxonomic units (OTUs) were created by clustering sequences with SILVA 16s V132 99% (at 97% similarity) used as the reference OTU database.

### 2.5 RNA extraction, library construction, sequencing, and data analysis

Total RNA extracted from Snap‐frozen mucosal biopsies using AllPrep DNA/RNA mini kit (Cat. 80204; Qiagen) following manufacturer's protocol and RNA integrity was determined by Aligent 2100 Bioanalyzer. Transcriptome libraries were prepared using TruSeq Stranded mRNA Library Prep kit (Illumina, Inc.) following the manufacturer's protocol and sequenced on the Novaseq System (Illumina, Inc.) with paired‐end (2 × 150 bp) reads. The reads were first mapped to the latest UCSC transcript set using Bowtie2 version 2.1.0 and the gene expression level was estimated using RSEM v1.2.15. Differentially expressed genes were identified using the edgeR program. Genes showing altered expression with *p* < 0.05 and more than 1.5‐fold changes were considered differentially expressed. ClusterProfiler was used for the Gene Ontology and pathway enrichment analysis.

### 2.6 Statistical analysis

Descriptive statistics were calculated, including medians, geometric means (95% confidence interval), as well as the ratio of geometric means (95% confidence interval) for each of the 15 analyzed BAs. Box plots were generated to depict the distribution of each BA by ethnicity. Generalized linear models with Gaussian family and log link regression analysis were used to compare the geometric means of BA levels between Caucasians and Hispanics. A *p*‐value <0.05 and a geometric mean ratio greater than or equal to 2.0 were considered statistically significant. Sensitivity analyses were performed by applying quantile regression analysis.

A two‐sample Wilcoxon rank‐sum (Mann–Whitney) test was also performed to compare the levels of all 15 BAs between male and female study participants. A *p*‐value <0.05 was considered significant.

OTU abundance of BA metabolizing bacteria at the levels of phylum, family, genus, and species was analyzed using Mann–Whitney *t*‐test. Measurements with a *p*‐value <0.05 were considered significant.

## 3 RESULTS

### 3.1 Characteristics of patients

Patient demographics and clinical characteristics were shown in Table 1. A total of 27 Caucasian and 20 Hispanic children and young adults diagnosed with mild to moderate UC were enrolled in the study. The average PUCAI score for Caucasians was 36.1 and for Hispanics was 42.8, with no significant difference between the two groups. The age range of the 47 patients was between 8 and 21 years old. Approximately 40% of the patients were males. 51.85% of Caucasians and 60% of Hispanics had a history of *C*. *difficile* infections showing no difference between the two groups. Patients were receiving one or more treatments for their UC (immunomodulators, biologics, steroids, 5‐aminosalicylates) at the time of stool sample collection, as shown in Table 1. There was no significant difference in the medications taken between our Hispanic and Caucasian cohorts. Food frequency questionnaires filled out by the study subjects did not reveal differences in nutrients or diet habits between the two groups.

**TABLE 1:** Patient characteristics.

	Caucasians	Hispanics	*p*‐Value
*N*	27	20	
Age—mean	16.0	17.2	0.14
Male gender—no. (%)	11 (40.7)	8 (40.0)	0.96
PUCAI score—mean	36.1	42.8	0.15
Mild disease—no. (%)	15 (55.6)	7 (35)	
Moderate disease—no. (%)	12 (44.4)	13 (65)	
History of *Clostridium difficile* infection—no. (%)	14 (51.85)	12 (60.0)	0.77
Medications for UC			
Proton pump inhibitor—no. (%)	0 (0.0)	2 (10.0)	0.16
Biologic—no. (%)	19 (70.4)	11 (55.0)	0.29
Immunomodulator—no. (%)	7 (25.9)	2 (10.0)	0.60
Steroid—no. (%)	4 (14.8)	4 (20.0)	0.66
5‐ASA—no. (%)	4 (14.8)	7 (35.0)	0.13

Abbreviations: 5‐ASA, 5‐aminosalicylates; PUCAI, Pediatric Ulcerative Colitis Activity Index.

### 3.2 Differences in BA profiles based on ethnicity

Fifteen conjugated and deconjugated BAs were extracted from each stool sample and analyzed in this study for comparison between the Caucasian and Hispanic groups. Those BAs included the two unconjugated PBAs (CA and CDCA) and their taurine and glycine conjugates, in addition to the three unconjugated SBAs (DCA, LCA, and UDCA) and their taurine and glycine conjugates as well.

**FIGURE 1:**
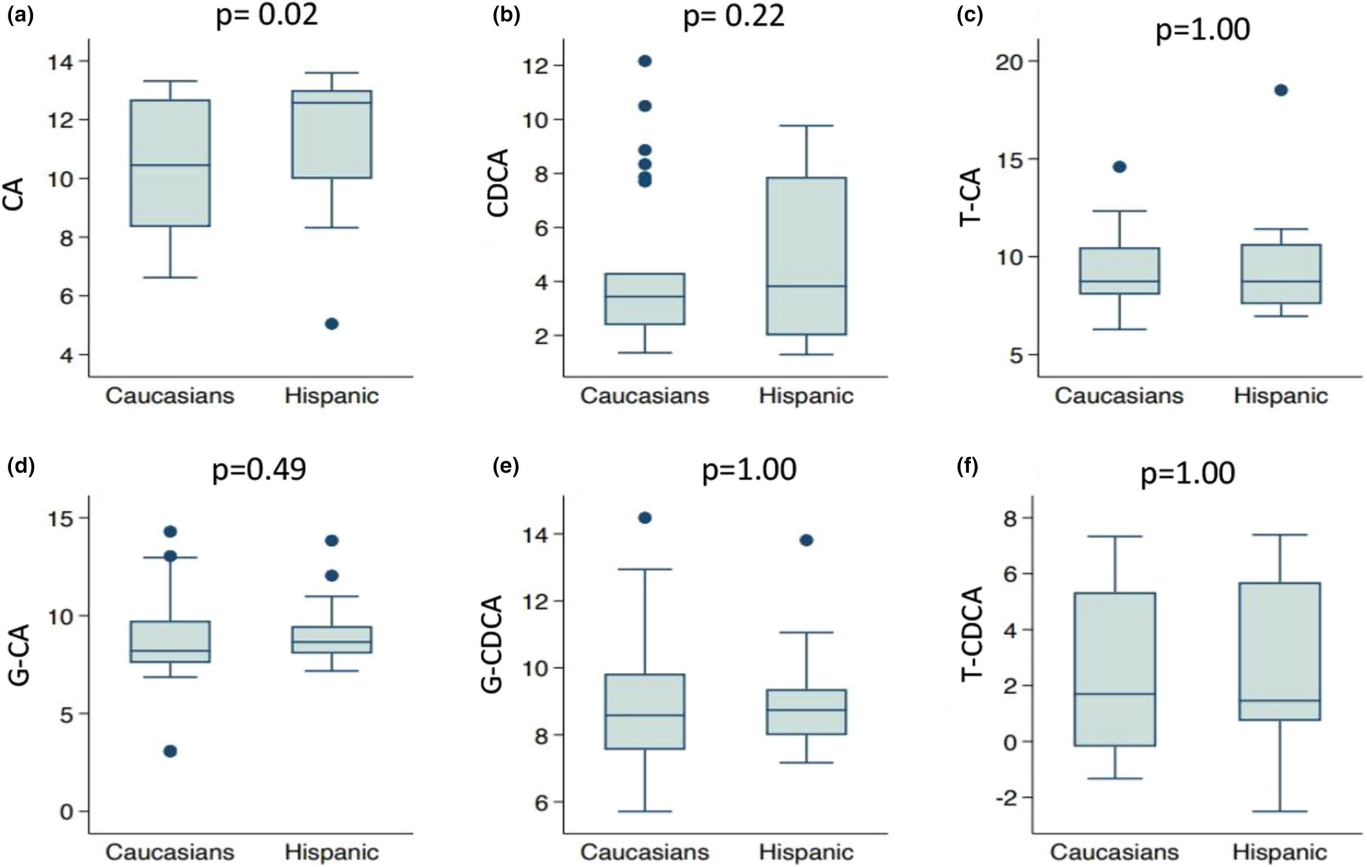
Boxplots of the log transformed concentrations (picograms/milligram feces) of the primary bile acids: cholic acid [(a) CA], chenodeoxycholic acid [(b) CDCA], taurocholic acid [(c) T‐CA], glycocholic acid [(d) G‐CA], glycochenodeoxycholic acid [(e) GDCA], and taurochenodeoxycholic acid [(f) T‐CDCA] in Caucasian and Hispanic subjects with ulcerative colitis.

**FIGURE 2:**
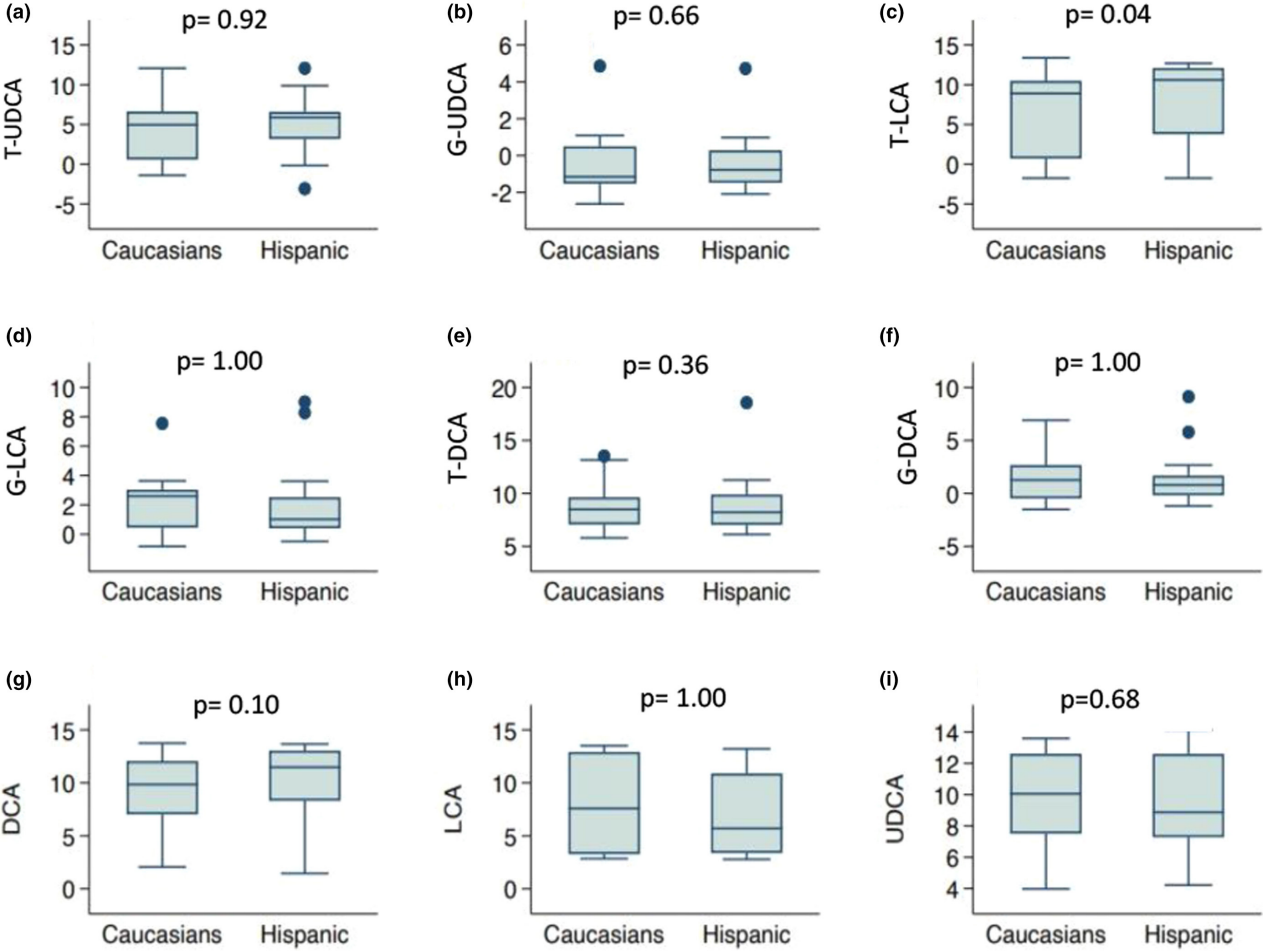
Boxplots of the log transformed concentrations (picograms/milligram feces) of the secondary bile acids: tauroursodeoxycholic acid [(a) T‐UDCA], glycoursodeoxycholic acid [(b) G‐UDCA], taurolithocholic acid [(c) T‐LCA], glycolithocholic acid [(d) G‐LCA], taurodeoxycholic acid [(e) T‐DCA], glycodeoxycholic acid [(f) G‐DCA], deoxycholic acid [(g) DCA], lithocholic acid [(h) LCA], and ursodeoxycholic acid [(i) UDCA] in Caucasian and Hispanic subjects with ulcerative colitis.

The primary BA, CA, showed the most significant difference between the two patient groups. The median of CA was significantly higher in Hispanic children and young adults with UC than their Caucasian counterparts (*p* = 0.02), as shown in Figure 1a. The ratio of the geometric means between the two groups showed that the geometric mean of CA was 2.1 times higher in Hispanics. CA's sensitivity analyses resulted in similar results, although with slightly different *p*‐values (results not shown). The median of the taurine conjugate of the SBA, LCA, was also significantly higher in Hispanic children and young adults (*p* = 0.04), as shown in Figure 2c. The ratio of geometric means between the two patient groups showed that the geometric means of T‐LCA were 4.7 times higher in Hispanics. Additionally, the median of the SBA, DCA, was found to be higher in Hispanics compared to Caucasians; however, it did not reach statistical significance (*p* = 0.10) (Figure 2g). The ratio of the geometric means between the two groups showed that the average level of DCA is 2.1 times higher in Hispanics than Caucasians.

The levels of the other 12 BAs analyzed in this study including T‐CA, G‐CA, CDCA, G‐CDCA, T‐CDCA, G‐DCA, T‐DCA, LCA, G‐LCA, UDCA, T‐UDCA, G‐UDCA did not differ significantly between the two groups (Figures 1 and 2). Geometric means of all BAs and *p*‐values for testing differences in BA medians between the two groups are shown in Table S1.

### 3.3 Differences in BA profiles based on gender

We also compared the levels of the 15 conjugated and deconjugated BAs between male and female pediatric patients included in this study. None of the BAs showed statistically significant difference between the two groups.

### 3.4 Differences in gut microbiota

**FIGURE 3:**
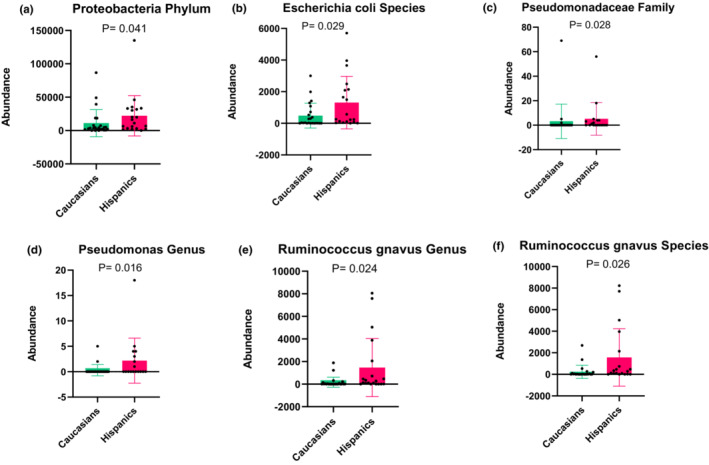
Bar plots of operational taxonomic unit abundance of Proteobacteria phylum (a), *Escherichia coli* species (b), Pseudomonadaceae family (c), *Pseudomonas* genus (d), *Ruminococcus gnavus* genus (e), *Ruminococcus gnavus* species (f) in Caucasian and Hispanic subjects with ulcerative colitis.

We compared the BA metabolizing gut microbiota between Caucasian and Hispanic children and young adults with UC at different taxonomic levels (Figure S1) to understand how they relate to shifting BA levels. Among the phyla that we found in the subjects' stool samples, we found that the abundance of the phylum Proteobacteria (Figure 3a), one of the most dominant phylum in IBD patients (Yang et al., 2021), was significantly higher in Hispanic subjects compared to Caucasians (22,151 vs. 11,084, *p* = 0.04) along with its *E*. *coli* (Figure 3b) species (1309.9 vs. 485.7, *p* = 0.03). When analyzing the bacterial abundance at the family level, Pseudomonadaceae (Figure 3c) was found to be significantly higher in Hispanic subjects (5.17 vs. 3.17, *p* = 0.03), consistent with the genus‐level results of significantly increased *Pseudomonas* (Figure 3d) in Hispanics than in Caucasians (1462.7 vs. 167, *p* = 0.02). Furthermore, comparing the abundance at the genus level showed that *R*. *gnavus* (Figure 3e) are significantly higher in Hispanics (196 vs. 7, *p* = 0.02). This finding was also consistent with the species of *R*. *gnavus* (Figure 3f) as it was found to be significantly increased in Hispanic subjects than in their Caucasian counterparts (1567.8 vs. 234.1, *p* = 0.03). None of the other intestinal bacteria that we found in the subjects' stool samples (Figure S1) showed a significant difference between Caucasians and Hispanics.

### 3.5 Differences in cytokine levels

**FIGURE 4:**
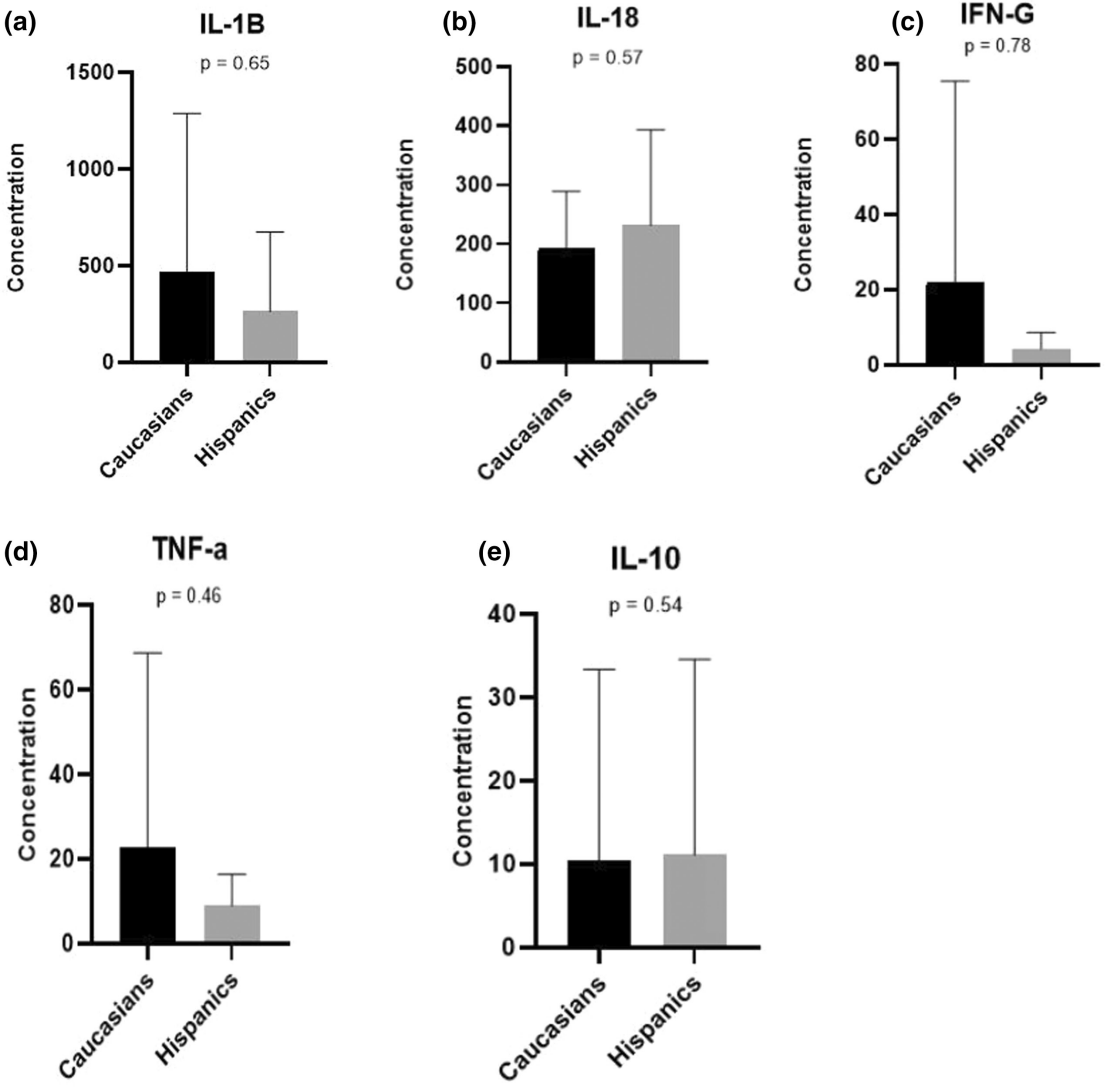
Boxplots of the concentrations (picogram/milligram protein) of cytokines: IL‐1B (a), IL‐18 (b), IFN‐G (c), TNF‐a (d), and IL‐10 (e) in Caucasian and Hispanic subjects with ulcerative colitis. IFN‐G, interferon‐gamma; IL, interleukin; TNF‐a, tumor necrosis factor‐alpha.

To understand if the gut microbiota differences that we found between Caucasians and Hispanics are related to the profiles of pro‐inflammatory and anti‐inflammatory cytokines, we compared the levels of interleukin (IL)‐1B, IL‐18, interferon‐gamma (IFN‐G), tumor necrosis factor‐alpha (TNF‐a), and IL‐10 between the two groups. Cytokines were extracted from representative colonic mucosal biopsies between 9 Caucasian and 13 Hispanic patients. Even though those cytokines did not show statistically significant difference in their levels between the two groups, there was a trend for the pro‐inflammatory cytokines (IL‐1B, IFN‐G, and TNF) being decreased in Hispanics, while the anti‐inflammatory cytokine, IL‐10, was similar in both groups as shown in Figure 4.

## 4 DISCUSSION

To the best of our knowledge, this is the first study describing differences in BAs and gut microbiota among two ethnic groups of pediatric UC patients. The profile of BAs and their metabolizing gut microbiota in Hispanic children and young adults with UC appears to be distinct from Caucasian children and young adults with UC.

Among the 15 BAs analyzed in this study, CA and T‐LCA were significantly higher in Hispanic children and young adults with UC. CA is one of the PBAs synthesized in the liver, and it has been reported to be increased in patients with IBD (Li et al., 2021). After CA gets secreted into the intestines, gut microbiota metabolizes it into different SBAs. T‐LCA, one of the conjugated SBAs, showed the second most significant difference between our two study groups. It has been reported that SBAs have an overall anti‐inflammatory properties, with LCA specifically providing a protective barrier in the large intestines that prevents the release of inflammatory factors (Shao et al., 2022). These findings indicate that our Hispanic patients have a BAs profile that provide them with protection from worsening inflammation. Differences in BAs were not influenced by differences in gender as comparing the levels of BA between male and female patients showed no difference between the two groups.

It has been well documented that the gut microbiota mainly regulates the process of synthesizing and metabolizing BAs (Ridlon et al., 2006). Diseases associated with gut dysbiosis, such as UC, can change the enzymatic reactions that transform PBAs to SBAs in the gastrointestinal tract. It has been reported that gut dysbiosis in IBD patients includes enrichment of the phylum Proteobacteria (Yang et al., 2021). Our findings show that the abundance of the microbiome that metabolizes BAs such as Proteobacteria is significantly higher in Hispanic subjects with UC than in their Caucasian counterparts. Moreover, the species of *E. coli* and *R. gnavus* have been reported to be enriched in UC patients (Berry & Reinisch, 2013; Hall et al., 2017; Yang et al., 2021). Our finding demonstrated that the abundance of both species is significantly higher in Hispanic subjects with UC when compared to their Caucasian counterparts. Gerard reported that *E*. *coli* plays a role in an enzymatic reaction that leads to generating SBAs (Gérard, 2013). Oxidation and epimerization reactions of PBAs are catalyzed by BA HSDH from intestinal bacteria, including *E*. *coli* species (Gérard, 2013; Sittipo et al., 2019). Moreover, The Ruminococcaceae family is known to have the ability to perform 7α‐dehydroxylation of CA and CDCA to generate SBAs (Sinha et al., 2020). Thus, the increased abundance of *E*. *coli* and *R*. *gnavus* found in our Hispanic children and young adults could contribute to higher SBA formation, which supports our finding of increased T‐LCA levels in that patient group. Additionally, *Pseudomonas* have been reported to catalyze desulfatation reactions producing SBAs (Gérard, 2013; Sittipo et al., 2019). This suggests that the higher levels of T‐LCA and DCA found in Hispanic children and young adults could also be partly due to the desulfatation reactions of the *Pseudomonas* genus that was found to be elevated in that patient group.

Since certain cytokines play an active role in inflammation by being pro‐inflammatory such as TNF‐a, IFN‐G, interleukin‐1B, and IL‐18, or anti‐inflammatory, such as IL‐10, we compared their levels between our Caucasian and Hispanic patients. Our results showed a trend of the pro‐inflammatory cytokine levels being lower in Hispanics as compared to Caucasians, which supports our hypothesis. However, our results did not reach statistical significance, perhaps due to the small sample size of colonic biopsies from which the cytokines were extracted.

Diet has been shown to impact the composition of the gut microbiome. Our food frequency questionnaire showed that Hispanic and Caucasian subjects in our study had similar diet histories, eliminating any dietary confounding factors that could have affected gut microbiota analysis. Given the fact that pediatric patients from both groups live in the US, this supports the finding of Damas et al. (2018) that most Hispanics make changes to their original diet when they immigrate to the United States. Most Hispanic patients with IBD in that study reported adoption of the American diet (Damas et al., 2018), which further explain why both groups in our study did not have major differences in their dietary habits.

It is important to note that this study had several limitations. Our sample size was small, and all patients were drawn from a single medical center. The BAs analyzed in this study were all extracted from one stool sample from each patient.

Overall, UC has been increasing in incidence and prevalence in the US among different racial and ethnic groups (Hou et al., 2009); however, most studies have focused on studying and describing the disease in the Caucasian population. Our analysis of fecal BAs and intestinal bacteria from pediatric patients diagnosed with UC demonstrated that children and young adults from different ethnic backgrounds could have different levels of BA and their metabolizing bacteria. Our findings in this study demonstrate that Hispanic children and young adults with UC have higher levels of CA and T‐LCA, in addition to, increased levels of intestinal bacteria including Proteobacteria, Pseudomonadaceae, Pseudomonas, *R*. *gnavus*, and *E*. *coli*, which suggest that they have a protective gut environment against inflammation. More research is needed to study differences in Hispanic and Caucasian children and young adults with UC since fixing dysbiosis and levels of key metabolites could be a promising therapeutic target for effective personalized UC treatment for patients from different racial groups.

## AUTHOR CONTRIBUTIONS

Lara Aboud Syriani interpreted the data, performed statistical analysis, and wrote the manuscript. Riddhi Parsana extracted bile acids, microbial DNA and RNA from collected samples, performed sequencing, and data analysis. Ramón A. Durazo‐Arvizu performed statistical analysis. Sonia Michail designed the study, supervised sample collection and processing, reviewed, and edited the manuscript. All authors approved the final version of this manuscript.

## FUNDING INFORMATION

This work was supported by the Biostatistics Core of The Saban Research Institute at Children's Hospital Los Angeles (CHLA) and by grants UL1TR001855 and UL1TR000130 from the National Center for Advancing Translational Science (NCATS). This work was also supported by the U.S. National Institutes of Health R01HD081197. The content is solely the responsibility of the authors and does not necessarily represent the official views of the National Institutes of Health.

## CONFLICT OF INTEREST STATEMENT

All authors have no conflict of interest to declare.

## ETHICS STATEMENT

This study was approved by the Institutional Review Board at the Children's Hospital of Los Angeles (IRB Study# CCI‐11‐148).

## Supporting information


Data S1.

Data S2.
