# Supplementary material for: Differences in gut microbiota and fecal bile acids between Caucasian and Hispanic children and young adults with ulcerative colitis
Source: Physiol Rep. 2023 Jun 21;11(12):e15752. doi: 10.14814/phy2.15752 (PMC10284820; doi:10.14814/phy2.15752)
Supplement: Supplementary file 1 — Data S1. [file PHY2-11-e15752-s002.pdf]

Table, Supplemental Digital Content 1. P-values for testing differences in medians and geometric means (95% confidence interval) per ethnic group for each of the 15 bile acids.

Bile Acid	P-value	GM_ Cauc	lb_GM_ Cauc	ub_ GM_ Cauc	GM_Hisp	lb_ GM_Hisp	ub_ GM_Hisp	GM_ratio	lb_ GM_ratio	ub_ GM_ratio
**CA**	0.02	193042.3	114241.7	326197.5	408094.8	276164.1	603052.4	2.1	1.1	4.0
**T-LCA**	0.04	25582.9	6157.4	106291.8	120812.6	75991.1	192071.1	4.7	1.1	20.5
**DCA**	0.10	99961.5	44710.1	223491.2	210543.2	112804.5	392966.6	2.1	0.9	5.1
**CDCA**	0.22	379.6	93.9	1533.5	914.8	333.1	2512.7	2.4	0.6	9.9
**T-DCA**	0.36	16.7	1.2	224.5	31.7	4.1	242.4	1.9	0.5	7.4
**G-CA**	0.49	4141.0	2238.8	7659.2	5269.2	2639.2	10520.2	1.3	0.6	2.5
**G-UDCA**	0.66	0.6	0.4	1.1	0.7	0.4	1.4	1.2	0.6	2.2
**UDCA**	0.68	131431.5	57963.9	298017.4	163571.3	68684.2	389544.9	1.2	0.4	3.5
**T-UDCA**	0.92	345.2	180.3	661.1	359.8	163.3	792.6	1.0	0.5	2.4
**G-LCA**	1.00	12.8	8.5	19.2	7.0	3.0	16.5	0.6	0.2	1.3
**G-CDCA**	1.00	5086.3	2998.8	8626.9	4232.1	2038.7	8785.5	0.8	0.5	1.5
**G-DCA**	1.00	3.7	1.7	7.7	2.3	0.9	6.0	0.6	0.3	1.4
**T-CDCA**	1.00	3128.1	694.3	14093.4	2013.7	364.6	11122.2	0.6	0.3	1.4
**T-CA**	1.00	9730.4	3741.7	25304.2	8115.4	2256.3	29188.9	0.8	0.4	1.9
**LCA**	1.00	12080.0	656.9	222138.3	6018.5	136.9	264622.0	0.5	0.1	2.7

Cholic acid (CA), glycocholic acid (G-CA), taurocholic (T-CA), chenodeoxycholic acid (CDCA), glycochenodeoxycholic acid (G-CDCA), taurochenodeoxycholic acid (T-CDCA), deoxycholic acid (DCA), glycodeoxycholic acid (G-DCA), taurodexoycholic acid (T-DCA), lithocholic acid (LCA), glycolithocholic (G-LCA), taurolithocholic acid (T-LCA), urosodeoxycholic acid (UDCA), glycoursodeoxycholic acid (G-UDCA), tauroursodeoxycholic acid (T-UDCA).

GM_ Cauc = Geometric Mean in Caucasians, lb_GM_ Cauc = Lower limit of 95% confidence interval for geometric mean among Caucasians, lb_GM_ Cauc = Upper limit of 95% confidence interval for geometric mean among Caucasians, GM_Hisp = Geometric Mean Hispanic, lb_ GM_Hisp = Lower limit of 95% confidence interval for geometric mean among Hispanics, ub_ GM_Hisp = Upper limit of 95% confidence interval for geometric mean among Hispanics, GM_ratio = ratio of geometric means: Caucasian Hispanics (gm1) to Caucasian(gm0), lb_ GM_ratio = lower limit of 95% confidence interval for geometric mean ratio, ub_ GM_ratio = upper limit of 95% confidence interval for geometric mean ratio.
