# Supplementary figures and images for: Differences in gut microbiota and fecal bile acids between Caucasian and Hispanic children and young adults with ulcerative colitis
Source: Physiol Rep. 2023 Jun 21;11(12):e15752. doi: 10.14814/phy2.15752 (PMC10284820; doi:10.14814/phy2.15752)

Figure, Supplemental Digital Content 2.

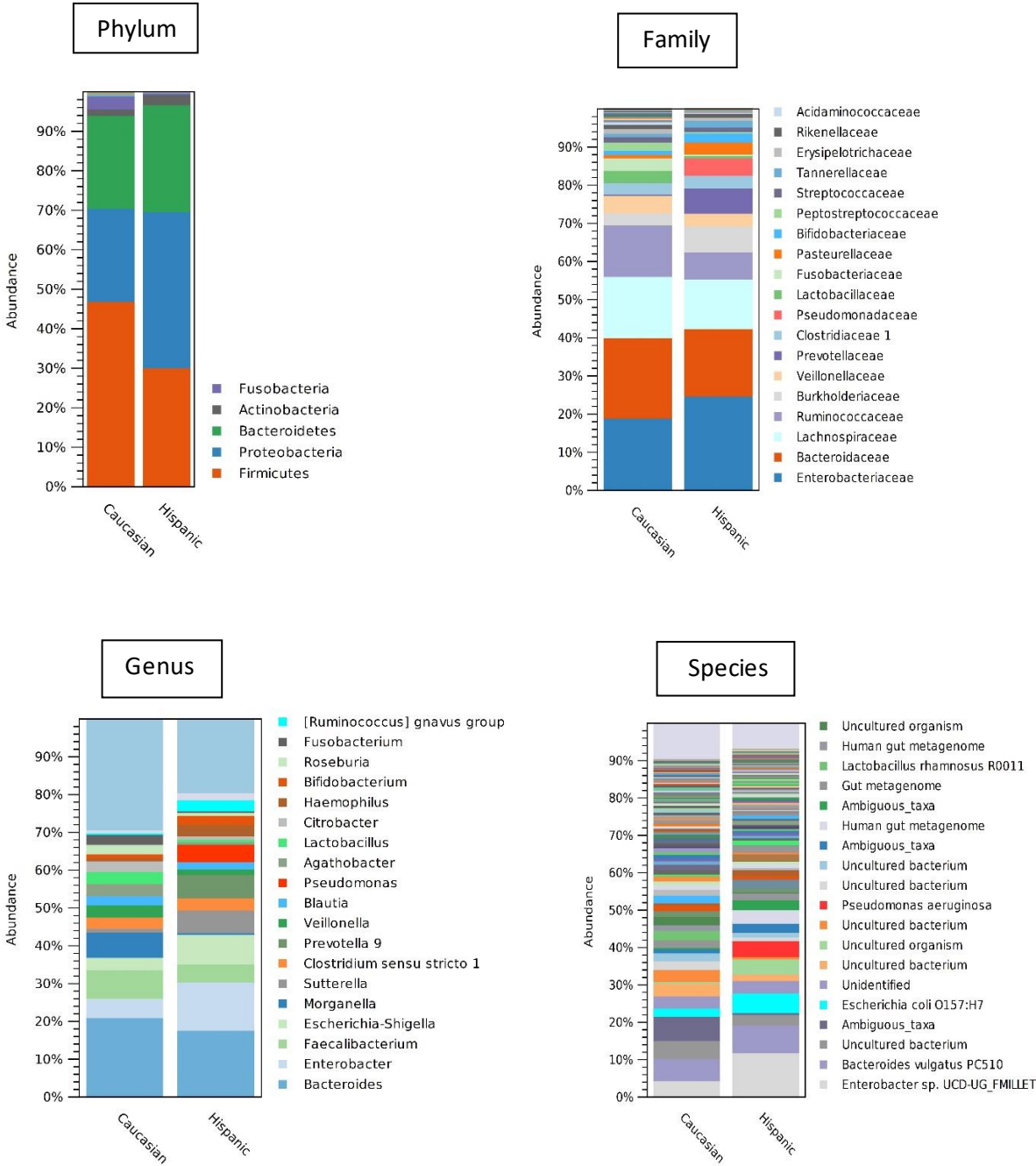

Supplement: Supplementary file 2 — Data S2. [file PHY2-11-e15752-s001.pdf]
